# The Rotunda Hospital, Dublin

**Published:** 1895-12-07

**Authors:** 


### THE ROTUNDA HOSPITAL DUBLIN.

### OPENING OF THE NEW WING.

The new wing of the Rotunda Hospital, commenced five years ago, and now completed at a cost of £11,000, was opened on Wednesday, November 27th, by Her Excellency the Countess Cadogan. The history of this hospital, "the oldest, largest, and most famous of those exclusively devoted to women in Her Majesty's dominions," to quote the words of the master's address on the occasion, is a brilliant record of 138 years' usefulness and progress. Its reputation is world-wide, nor is its prestige at the present day one whit diminished. And Ireland is justly proud of the famous hospital which has done so much to shed lustre on Irish medical science, and the fame of which attracts to Dublin every year numbers of fully-qualified medical men, not only from England and Scotland, but from the Continent, America, and Australia, who feel their education incomplete until they have added the experience to be gained within its walls. It is, therefore, a matter of general rejoicing here that the new additions and improvements, which will add so much to the efficiency of the hospital, are at length completed. These consist in a wing of three stories, the ground floor being occupied by consulting and examining rooms, dispensary, waiting-room, isolation ward, small wards for paying patients, sisters' sitting-room, ward maids' quarters, &c., and the first floor by two large wards, 65 by 25 feet, each containing 16 beds, and some smaller wards, which will all be devoted to the reception of surgical cases, the old building giving ample accommodation to the maternity patients. A new operation theatre is also placed on this floor, that portion set apart for pupils being divided from the rest by a lass partition, through which, of course, any operation in progress can be distinctly seen, while at the same time the air around the patient is kept as far as possible pure and undisturbed. The entire third story is devoted to the nurses' dormitories, and the accommodation provided for them leaves nothing to be desired. A hydraulic lift, so placed as to be of equal use to both the old and new portions of the hospital, is not the least of the new improvements, and all the sanitary arrangements, which include due accommodation for the disinfection of soiled linen and the destruction of refuse, are also easily accessible from both wings. That portion of the old building now vacated by patients will be utilised as a residence for pupils. The opening ceremonial took place in one of the large wards of the new wing, their Excellencies Lord and Lady Cadogan being received upon their arrival and conducted thither by the Master of the Hospital (Dr. Smyly), the Right Hon. the Lord Mayor, and several of the governors. A beautiful bouquet having been presented to the Countess on behalf of the nursing staff, and a short address read by Dr. Smyly, her Excellency declared the new wing open for the reception of patients, naming it, as requested, "The Thomas Plunkett Cairns Wing of the Rotunda Hospital." A few other speeches followed, including an excellent and sympathetic one from the Lord Lieutenant, after which the Viceregal party proceeded to the Round Room of the Rotunda, and formally opened "The Winter Garden Fête," which has been inaugurated as a first step towards the removal of the debt in which the heavy, but necessary, outlay on the new building has involved the hospital.

